# Epidemiology and Molecular Characterization of *Cryptosporidium* spp. in Humans, Wild Primates, and Domesticated Animals in the Greater Gombe Ecosystem, Tanzania

**DOI:** 10.1371/journal.pntd.0003529

**Published:** 2015-02-20

**Authors:** Michele B. Parsons, Dominic Travis, Elizabeth V. Lonsdorf, Iddi Lipende, Dawn M. Anthony Roellig, Shadrack Kamenya, Hongwei Zhang, Lihua Xiao, Thomas R. Gillespie

**Affiliations:** 1 Program in Population Biology, Ecology, and Evolution and Departments of Environmental Sciences and Environmental Health, Emory University, Atlanta, Georgia, United States of America; 2 Division of Foodborne, Waterborne, and Environmental Diseases, Centers for Disease Control and Prevention, Atlanta, Georgia, United States of America; 3 College of Veterinary Medicine, University of Minnesota, Minneapolis, Minnesota, United States of America; 4 Department of Psychology, Franklin and Marshall College, Lancaster, Pennsylvania, United States of America; 5 The Jane Goodall Institute, Kigoma, Tanzania,; 6 Institute of Parasite Disease Prevention and Control, Henan Center for Disease Control and Prevention, Zhengzhou, China; Oxford University Clinical Research Unit, VIETNAM

## Abstract

*Cryptosporidium* is an important zoonotic parasite globally. Few studies have examined the ecology and epidemiology of this pathogen in rural tropical systems characterized by high rates of overlap among humans, domesticated animals, and wildlife. We investigated risk factors for *Cryptosporidium* infection and assessed cross-species transmission potential among people, non-human primates, and domestic animals in the Gombe Ecosystem, Kigoma District, Tanzania. A cross-sectional survey was designed to determine the occurrence and risk factors for *Cryptosporidium* infection in humans, domestic animals and wildlife living in and around Gombe National Park. Diagnostic PCR revealed *Cryptosporidium* infection rates of 4.3% in humans, 16.0% in non-human primates, and 9.6% in livestock. Local streams sampled were negative. DNA sequencing uncovered a complex epidemiology for *Cryptosporidium* in this system, with humans, baboons and a subset of chimpanzees infected with *C. hominis* subtype IfA12G2; another subset of chimpanzees infected with *C. suis*; and all positive goats and sheep infected with *C. xiaoi*. For humans, residence location was associated with increased risk of infection in Mwamgongo village compared to one camp (Kasekela), and there was an increased odds for infection when living in a household with another positive person. Fecal consistency and other gastrointestinal signs did not predict *Cryptosporidium* infection. Despite a high degree of habitat overlap between village people and livestock, our results suggest that there are distinct *Cryptosporidium* transmission dynamics for humans and livestock in this system. The dominance of *C. hominis* subtype IfA12G2 among humans and non-human primates suggest cross-species transmission. Interestingly, a subset of chimpanzees was infected with *C. suis*. We hypothesize that there is cross-species transmission from bush pigs (*Potaochoerus larvatus*) to chimpanzees in Gombe forest, since domesticated pigs are regionally absent. Our findings demonstrate a complex nature of *Cryptosporidium* in sympatric primates, including humans, and stress the need for further studies.

## Author Summary

*Cryptosporidium* is a common zoonotic gastrointestinal parasite. In a cross-sectional survey of humans, non-human primates (chimpanzees and baboons) and livestock in the Greater Gombe Ecosystem, Tanzania, *Cryptosporidium* infection rate was 4.3%, 16.0% and 9.6% respectively. Infection was not associated with clinical disease in people; however, living in a household with an infected person increased one’s risk of infection. Phylogenetic analyses identified clusters of *Cryptosporidium* with a mixed host background. Surprisingly, the Mitumba chimpanzee community, which shares a natural boundary with a human community, had a lower occurrence of *C*. *hominis* compared to the Kasakela chimpanzee community, which resides in the forest interior (less human exposure). However, Kasakela chimpanzees were also infected with *C*. *suis*, suggesting a transmission cycle linked to sympatric bush pigs. Our findings highlight the complex nature of zoonotic parasite transmission and stress the need for further studies in similar systems.

## Introduction

*Cryptosporidium* is one of the most important parasitic diarrheal agents in humans in the world, is among the top four causes of moderate-to-severe diarrheal disease in young children in developing nations, and is problematic as an opportunistic co-infection with HIV due to increased morbidity and mortality [1,2]. *Cryptosporidium* is well adapted to zoonotic, waterborne, and foodborne transmission, with a life cycle occurring in suitable hosts and transmission by the fecal-oral route [3]. Zoonoses represent the majority of diseases emerging globally with potential to expand to new host systems, [4] yet despite these health threats, few studies have examined the ecology and epidemiology of this pathogen in rural tropical forest systems characterized by high rates of overlap among humans, domesticated animals, and wildlife [5,6].

In Tanzania, agriculture represents over a quarter of the national income and 80 percent of its labor force [7], but natural resources are declining, affected by desertification and soil degradation from recent droughts. This process has resulted in a high rate of loss of forest and woodland habitat [8]. The resulting fragmented landscape increases human-wildlife contact in these areas, elevating the risk for disease transmission. The Greater Gombe Ecosystem (GGE), Tanzania, in particular, is vulnerable to habitat disturbance, and this has both ecological and financial implications since it is home to diverse wildlife, including endangered chimpanzees (*Pan troglodytes schweinfurthii*), that are important contributors to the national economy through tourism [7].

Gombe National Park, established in 1968, is a small 35-km^2^ forest reserve located 16-km north of Kigoma in Western Tanzania (4°40′S 29°38′E). The park is 1500-m above sea-level with hills sloping westward from a rift escarpment to Lake Tanganyika [9]. It is home to a number of non-human primate species, including baboons (*Pabio anubis*), and a well-known wild chimpanzee population studied continuously for over 50 years [10,11]. There are three chimpanzee communities (Kasekela, Mitumba and Kalande); two of which, (Kasekela, and Mitumba) are habituated [12]. The habitat ranges of these two communities overlap slightly permitting opportunity for member contact. Their habitats have differing degrees of human encroachment [13]. Kasekela, the larger community (∼ 65 individuals), is situated at the center of the park in less disturbed forest, whereas Mitumba, the smaller Northern community (∼ 25 individuals), is in close proximity to Mwamgongo (4°40′S, 29°34’60′ E), a village home to ∼5000 inhabitants and their livestock. Another village borders the park to the South, but not along the Eastern ridge, due to high elevation and historic soil depletion. Human presence in the park is limited to researchers, tourists, park management staff, local field assistants and members of their families. The park border is not fenced and therefore villagers and their untethered animals (goats, sheep and dogs) are able to enter the park [14]). Mitumba chimpanzees are frequently reported raiding agricultural fields to the east in the Northern village, Mwamgongo, especially during the dry season (I. Lipende, personal communication). There is little evidence that the chimpanzee population has emigrated outside its established habitat for over 20 years, and immigration events are rare.

Death from infectious diseases is the leading cause of mortality for Gombe chimpanzees [15,16]. The chimpanzees have experienced SIVcpz-associated mortality and morbidity, with SIVcpz prevalence ranging between 9–18% and a 10–16-fold higher age-corrected death hazard for infected individuals [17]. *Cryptosporidium* is of special concern in this chimpanzee population, as SIVcpz illness may be complicated by *Cryptosporidium* co-infection, and mirror clinical features observed in human HIV/*Cryptosporidium* co-infections [2], that report *Cryptosporidium* infection rates from 8–30% [18,19]. To improve our understanding of this relationship, and highlight potential management options, we investigated risk factors for *Cryptosporidium* infection and assessed cross-species transmission potential among people, non-human primates, and domestic animals in the GGE, Kigoma District, Tanzania.

## Materials and Methods

### Ethics statement

This project was reviewed and approved by the Emory University Institutional Review Board (approval #: IRB00018856) under the Expedited review process per 45 CFR 46.110(3), Title 45 CFR Subpart D section 46.404, one parent consent, and 21 CFR 56.110 and the Tanzanian National Institute for Medical Research Institute, Dar Es Salaam, Tanzania, which approved oral consent due to low literacy rates. All adult subjects provided informed consent, and a parent or guardian of any child participant provided informed consent on their behalf. Oral informed consent was obtained by trained local field assistants and documented by witnessed notation on IRB-approved enrollment forms. All animal use followed the guidelines of the Weatherall Report and the NIH Guide for the Care and Use of Laboratory Animals on the use of non-human primates in research, and was approved by the Tanzania Wildlife Research Institute and Tanzania Commission for Science and Technology (permit number 2009-279-NA-2009-184), and the Emory University Animal Care and Use Committee (protocol ID 087-2009). Approval was also obtained from Tanzania National Parks (Permit number TNP/HQ/C10/13) to collect samples from wild chimpanzees. The researchers did not have any interactions with the chimpanzees in the park. All domesticated animals were sampled from households in Mwamgongo village. The owners of the domesticated animals provide verbal consent for the collection of fecal specimens for this study, and the verbal consent was documented. We have included the GPS coordinates for Mwamgongo village at the first mention of the village in the Introduction.

### Sample frame

The study period occurred between March 2010 and February 2011. Paired fecal samples from humans and domestic animals were collected during the dry (July 1-August 15) and wet (November 1-December 15) seasons. Human subjects were either residents of Mwamgongo village (estimated population size (n) ∼5000) or Gombe National Park (n ∼100). A baseline demographic survey was performed in June 2010 to identify households within Mwamgongo village with at least one domestic animal species: dog (*Canis lupus*) n ∼ 8, goat (*Capra hircus*), n ∼ 150 or sheep (*Ovis aries*), n ∼ 10. Twenty-five village households with domestic animals were randomly selected for study enrollment. Baboons (n ∼ 198) were opportunistically sampled in Mitumba and Kasekela during these two collection periods. Chimpanzees (n ∼ 90) were sampled in both Mitumba and Kasekela at quarterly intervals during the course of routine observational health monitoring [16].

### Specimen collection and transport

Specimen cups were provided to enrolled village and park residents. Livestock specimens were aseptically collected by a village veterinary officer. Chimpanzee and baboon specimens were non-invasively collected from identified individuals as part of observational health monitoring. All fecal specimens were freshly voided and aseptically transferred to a screw cap plastic vial containing a 2.5% potassium dichromate solution (Fisher Scientific, Pittsburgh, PA). For baboon and non-human primate samples, care was taken to avoid the collection of soil, foliage or water contaminants, by transferring the interior and top most portion of stool to a collection cup using a sterile wooden spatula or swab and avoiding the collection of fecal material in contact with the ground. Each vial was labeled with a unique identification number, and date of collection. Wildlife samples were additionally labeled with the name of the observer, location and animal name. Samples were sealed with Parafilm (Pechiney Plastic Packaging, Chicago, IL) and stored at 4°C, and shipped in ice to Atlanta, GA United States.

### Stream sampling

Approximately 1-liter of water was collected in 55-oz sterile Whirl-pak bags (Nasco, Fort Atkinson, WI) and filtered for protozoa using a 0.45-μm Millipore MF-Millipore cellulose ester filter mounted on the Millipore (Billerica, MA) filtration system (diameter 47-mm). When possible, filtration was done on one filter, but in extreme cases where turbidity was high, sequential filtration was performed using two filters. Using sterilized forceps, filters were aseptically transferred to 2-ml cryovials containing a 2.5% potassium dichromate solution. Due to the logistics of field sampling, opportunistic water samples were collected from low, middle and high points of 6 continual streams (dry and wet) and two seasonal streams (wet only). GPS coordinates were obtained using a GPSmap 60CSx from Garmin (Garmin International Inc. Olathe, KS) for each collection point to assist in identifying locations for repeat sampling and if necessary, to assign sampled streams to watersheds associated with specific human or chimpanzee groups.

### DNA extraction, molecular detection and subtyping

Nucleic acid was extracted from all fecal specimens and water filters using the FastDNA® SPIN Kit for Soil (MP Biomedicals, LLC, Solon, OH) following the methods described [20]. DNA extracts were subsequently tested using a polymerase chain reaction and restriction fragment length polymorphism (PCR-RFLP) approach where a segment (∼833 bp) of the *Cryptosporidium* SSU rRNA gene is amplified by nested PCR and then species and genotype diagnosis is made by restriction digestion of the secondary PCR product with *Ssp*I (New England BioLabs, Beverly, MA), and either *Vsp*I (Promega, Madison, WI) or *Mbo*II (New England BioLabs) [21,22]. Each sample was run in duplicate by PCR-RFLP analyses with appropriate controls. Specimens that were positive for *Cryptosporidium* by the SSU rRNA PCR were confirmed by DNA sequencing of the 18S PCR products (*C*. *suis*, *C*. *xiaoi and C*. *hominis*). A subset of specimens positive for *C*. *hominis* were also subtyped by sequencing the 60-kilodalton glycoprotein (GP60; ∼900 bp) in both directions on an ABI 3130 Genetic Analyzer (Foster City, CA) [23]. All sequences obtained were aligned with reference sequences using MEGA 6.0 or ClustalX software (http://www.clustal.org/) to identify *Cryptosporidium* species and *C*. *hominis* genotypes.

### Human risk factor survey

A survey was administered to each human subject focusing on demography, gastrointestinal symptoms (presence or absence within the previous 4 weeks), medication usage, and water usage. To minimize response bias, surveys were administered by trained local field assistants in the national language (Swahili). Data were manually recorded on paper forms, entered into spreadsheets in the computer program Microsoft Excel, and subsequently reviewed for accuracy.

### Statistical analyses and control for sample bias

Results were tabulated and compared in Microsoft Excel (Redmond, WA). To control for sample bias we calculated infection rate as the proportion of individuals in each group positive for *Cryptosporidium* divided by the total number of individuals in each group examined [24]. If a single individual sample was positive for *Cryptosporidium*, the subject was considered positive for the collection period (the season for most analyses). Statistical analyses were performed in SPSS version 20.0 (SPSS Inc., Chicago IL). Associations between human survey responses and infection status were compared using logistic regression for categorical binary data. Work history (agricultural fields or forest) were combined as a single factor in the final analysis. Odds ratios (OR) with 95% CI were calculated with significance set at 0.05 for all comparisons. Associations between chimpanzee demographic and observational health data and infection status were evaluated using a generalized estimating equation (GEE) method with exchangeable working correlation structure to account for repeat sampling of individuals. The Huber–White sandwich variance estimation technique was used to calculate confidence intervals (CI). In instances where cells contained less than 5 values, Fisher’s exact tests were used to calculate *p*-values.

## Results

Six hundred and eighty-four fecal specimens were screened for *Cryptosporidium* including 254 human, 99 domestic animal (n = 76 goat, n = 14 sheep, n = 9 dog) and 331 wildlife (n = 251 chimpanzee, n = 80 baboon) specimens. *Cryptosporidium* spp, were detected by PCR from 40 (5.8%) fecal samples but was not detected in any water samples (n = 42). The infection rate of *Cryptosporidium* was highest among 21/131 (16.0%) nonhuman primates tested, compared to 7/73 (9.6%) livestock and 8/185 (4.3%) humans. No significant differences in frequency were observed between chimpanzees and baboons (Table 1, Fisher’s exact test *p* = 0.457) or between the two chimpanzee communities (Table 1, Fisher’s exact test *p* = 0.7655). Of the 8 cases of *Cryptosporidium* detected in humans, 7 (87.5%) resided in Mwamgongo village and one (12.5%) in Mitumba camp. No human cases were detected in the Kasekela camp. Sheep had the highest occurrence of *Cryptosporidium* (22%) compared to goat (9%) and dogs (0%) but small sample size prevented evaluation of significance.

**Table 1:** Infection rate of *Cryptosporidium* species and *C*. *hominis* subtypes detected by species and location in and around Gombe National Park, Tanzania.

Host	Positive/Total	*Cryptosporidium* species detected (n)	*C*. *hominis* genotypes	Infection Rate (95% CI)
**Humans**				
Mwamgongo Village	7/95	*C*. *hominis* (7)	IfA12G2 (6/7)	0.07 (0.03–0.15)
Kasekela	0/58			0.0 (0.00–0.08)
Mitumba	1/32	*C*. *hominis* (1)	IfA12G2 (1/1)	0.03 (0.03–0.15)
**Livestock**				
Dog	0/8			0 (0.00–0.40)
Goat	5/56	*C*. *xiaoi* (5)		0.09 (0.03–0.20)
Sheep	2/9	*C*. *xiaoi* (2)		0.22 (0.03–0.60)
**Non-human primates**				
Baboon	5/47	*C*. *hominis* (5)	IfA12G2 (3/5)	0.11 (0.04–0.24)
Kasekela Chimpanzees				
All *Cryptosporidium*	12/58			0.21 (0.11–0.34)
*C. hominis*	6/58^a^	*C*. *hominis*(7)	IfA12G2 (2/7)	0.10 (0.04–0.22)
*C. suis*	7/58^a^	*C*. *suis* (6)		0.12 (0.05–0.23)
Mitumba Chimpanzees	4/26	*C*. *hominis* (4)	IfA12G2 (3/4)	0.15 (0.05–0.36)

^a^One individual was positive for both *C*. *suis* and *C*. *hominis*

We identified three species of *Cryptosporidium* (*C*. *hominis*, *C*. *suis* and *C*. *xiaoi*) in this population (Table 1) based on RFLP and sequence analyses of the SSU rRNA gene. *C*. *hominis* was detected in all human cases and all 7 cases from sheep and goats were *C*. *xiaoi*. Six of the 12 positive chimpanzees from Kasekela were genotyped as *C*. *suis* (a *Cryptosporidium* species predominantly associated with pigs but has been found in a few human cases). This species was not detected in the Mitumba chimpanzee community (Fisher’s two-tailed exact test; *p*-value = 0.0537). The porcine species was also not found in the specimens from baboons, humans or domestic animals in the village. The remaining Kasekela chimpanzees (n = 6) and the 4 Mitumba chimpanzees had *C*. *hominis*. All baboons (n = 5 individuals) also had *C*. *hominis*. GP60 subtyping of a subset (n = 16) of the *C*. *hominis* positive samples identified a common subtype IfA12G2 in humans and nonhuman primates. The subtype sequence was identical to two sequences in GenBank; a human *C*. *hominis* IfA12G2 sequence from South Africa (GenBank accession number JN867334) and a sequence recovered from an olive baboon in Kenya (GenBank accession number JF681172).

We used data from the survey to identify potential risk factors for *Cryptosporidium* infection (Table 2). Among 95 respondents (100%), villagers in Mwamgongo were at greater risk for infection when living with a person who was positive for *Cryptosporidium* (OR = 9.722; 95% CI 1.741–54.279; *p* = 0.011). Persons living with *Cryptosporidium*-positive livestock tended to have a greater odds of infection (OR = 4.750; 95% CI 0.944–23.908; *p* = 0.059). Other factors related to behaviors, including location, occupation (either agricultural or forestry), and not boiling water for consumption were not statistically significant. Although presence of clinical signs was not statistically significant, when reviewing survey data for the *Cryptosporidium* positive patients, 4/8 reported having diarrhea; 2 sought treatment at the village clinic (Flagyl and Paracetemol). Four of 8 households (50%) reported at least one additional member of the household experiencing gastrointestinal symptoms, including diarrhea and cramping. Interestingly, affected individuals did not report consuming water from an open source (100%), which appeared protective (OR = 0.156; 95% CI 0.085–2.875) but not statistically significant (*p* = 0.162). 4% of study respondents reported boiling their water before use. No infected individuals reported boiling their drinking water. An association between season and *Cryptosporidium* infection was not observed in humans or non-human primates (Tables 2 and 3). Chimpanzee demographic factors such as age and sex were not risk factors for *Cryptosporidium* infection and evidence of diarrhea was not a reliable predictor of *Cryptosporidium* illness. Kasekela chimpanzees tended to have a higher likelihood (OR = 7.062; 95% CI 0.398–125.251, *p* = 0.07) of infection with *C*. *suis* as compared to the Mitumba community (Table 3).

**Table 2:** Risk factors for *Cryptosporidium* infection in people living in or adjacent to Gombe National Park, Tanzania.

			95% CI		
Variable	n	OR	Lower	Upper	p
Age (≤7 years)	179	0.834	0.096	7.277	0.870
Sex (female vs. male)	184	0.946	0.378	2.368	0.905
Seasonality: dry vs wet season	185	2.044	0.244	17.107	0.510
Location (Mwamgongo vs Mitumba)	127	0.406	0.048	3.429	0.407
Location (Mitumba vs Kasekela)	90	5.571	0.2204	140.820	0.356
**Location (Kasekela vs Mwamgongo)**	**153**	**9.915**	**0.556**	**176.950**	**0.045**
Mwamgongo vs park resident	185	7.080	0.853	58.740	0.070
Experienced gastrointestinal symptoms	147	1.458	0.256	8.301	0.671
Used commercial or traditional medicine	173	0.554	0.03	10.194	0.473
Consumption of water from open water source	163	0.156	0.0085	2.875	0.162
Work in agricultural fields or forest	171	3.529	0.665	18.730	0.139
Water not boiled before consumption	153	1.990	0.0992	40.028	0.816
**Live in household with another positive person (Mwamgongo)**	**95**	**9.722**	**1.741**	**54.279**	**0.011**
Live in household with positive livestock (Mwamgongo)	95	4.750	0.944	23.908	0.059

**Table 3:** Risk factors for *Cryptosporidium* infection in chimpanzees in Gombe National Park, Tanzania.

Variable		All *Cryptosporidium*				*C. suis*				*C. hominis*			
			95% CI				95% CI				95% CI		
	n^a^	OR	Lower	Upper	p	OR	Lower	Upper	p	OR	Lower	Upper	p
Age (≤10 years)	84	1.160	0.395	3.411	0.788	1.950	0.413	9.207	0.399	0.737	0.182	2.992	0.670
Sex (female vs. male)	84	0.425	0.128	1.412	0.162	0.181	0.022	1.504	0.113	0.716	0.172	2.980	0.647
Seasonality: (dry vs wet)	84	1.194	0.440	3.245	0.728	0.875	0.226	3.391	0.848	1.471	0.459	4.745	0.514
Location (Kasekela vs Mitumba)	84	0.935	0.274	3.187	0.915	7.062	0.398	125.251	0.070	2.354	0.616	8.998	0.211
Observed to have diarrhea	79	2.034	0.703	5.883	0.190	1.461	0.264	8.069	0.664	2.425	0.632	9.309	0.197

^a^Sample sizes may vary based on number of individual observations.

Binary logistic regression was used to calculate odds ratios, confidence interval and significance in most cases. Fisher’s Exact test was used to calculate *p*-values when cells contained values less than 5.

## Discussion

Of 90 humans residing in camps within Gombe National Park, only one *Cryptosporidium* infection was observed (1%). In contrast, *Cryptosporidium* infection in residents of Mwamgongo village reached 10% during the drier months. These frequencies are comparable to those reported in some studies from children and adults without HIV, ranging from 0–18% [25–27], though frequencies as high as 32% have been reported elsewhere [28]. Site and HIV prevalence would seem to be important factors in human occurrence rates. Although HIV testing was beyond the scope of this study, a recent country report [9] indicates a low HIV prevalence (< 1%) from the Kigoma region where the study occurred. Infection was not statistically associated with gastrointestinal illness or stool consistency for humans or wildlife, findings consistent with earlier studies [28–32]. Surprisingly, unsafe drinking water (i.e. untreated/unboiled water, open water source) was not found to increase risk of *Cryptosporidium* infection, which could be the result of degradation or improper tapping of the water line, exposing water to environmental contamination [33]. This warrants further study as consumption of contaminated ground water has been repeatedly associated with *Cryptosporidium* infection [34,35].

*Cryptosporidium* was detected in baboons (11%) and the two chimpanzee communities (15–21%) at moderate frequencies. Similar frequencies of *Cryptosporidium* have been detected previously in other African primates. Habituated mountain gorillas in Bwindi Impenetrable National Park (BINP), Uganda had *Cryptosporidium* in (11%) of specimens sampled; 73% of positive specimens were detected from human-habituated gorillas [36]. Genetic characterization of this population determined that the gorillas and the local human community both carry *C*. *parvum* [37]. Eleven percent of red colobus and black-and-white colobus in Kibale National Park (KNP), Uganda were infected with *Cryptosporidium*; genetic sequences from some humans and colobus from KNP were identical, while two strains from red colobus in the forest interior were infected with a divergent subclade, suggesting the possibility of separate zoonotic and sylvatic cycles [28]. These findings support the zoonotic spillover potential of *Cryptosporidium* among humans, wildlife and livestock.

Similar patterns have been observed with other directly or environmentally transmitted enteric pathogens. At KNP, *Giardia* was detected in red colobus in forest fragments (5.7%), but not detected in undisturbed forest (0%) [38]. Genetic analyses of recovered strains determined that red colobus were infected with *Giardia duodenalis* assemblages associated with humans and livestock, suggesting complex cross-species transmission [39]. *Escherichia coli* strains from gorillas in BINP and chimpanzees in KNP that overlapped with humans were more similar to strains collected from resident humans and livestock compared to strains collected from gorillas and chimpanzees living in undisturbed forest [40,41]. Additionally, in KNP, genetic similarity between human/livestock and primate bacteria increased three-fold with a moderate to high increase in anthropogenic disturbance of forest fragment. Bacteria harbored by humans and livestock were more similar to those of monkeys that entered human settlements to raid crops, than to bacteria of other primate species [42]. These findings reinforce the notion that habitat overlap and anthropogenic disturbance increase the risk of interspecies transmission between wildlife, humans and livestock and that transmission can occur both via direct physical contact with or ingestion of contaminated feces and by indirect exposure via a shared (potentially contaminated) watershed. Although water samples screened in this study were negative for the parasite, waterborne outbreaks of *Cryptosporidium* as a result of human and animal fecal contamination are common [43–45]. Watershed sampling for *Cryptosporidium* in this study was opportunistic using smaller volumes of water than advocated by standard screening protocols [46] and provided only limited inter-seasonal sampling [47–49]. Therefore, our negative results do not assure that waterborne transmission is not important in this system. Future studies using more comprehensive watershed sampling would help to resolve this aspect of *Cryptosporidium* transmission.

The results of our RFLP and sequence analyses of the SSU rRNA gene suggest multiple potential zoonotic pathways for *Cryptosporidium* transmission in this study system. The village data reinforces our understanding that species of *Cryptosporidium* vary in their zoonotic potential [50]. Our data suggest that there is less likelihood that the *C*. *xiaoi* affecting the livestock is capable of causing illness in humans, considering the close proximity of livestock to humans in this community [i.e., animals often residing in homes], where animal-human contact is quite high though it has been documented in other studies [51]. *Cryptosporidium hominis* was also not detected among the domesticated animals in this study, but *C*. *hominis* has been found in other studies to be a zoonotic species, affecting both humans and domesticated animals [52,53]. Homes with positive livestock had a tendency for increased risk of human infection suggesting contribution of environmental factors or behaviors that may place the household at increased risk.

We anticipated that the Kasekela chimpanzee community would have a lower occurrence of *Cryptosporidium* compared to Mitumba, which shares a natural border with Mwamgongo village. Although not statistically significant, there was a higher frequency of *Cryptosporidium* recovered in Kasekela. The occurrence of *C*. *hominis* was not statistically higher for Mitumba compared to Kasekela. However, the results demonstrated that 50% (6/12) of the *Cryptosporidium* recovered from the Kasekela community are *C*. *suis*, a species associated with pigs. Domestic pigs are not found in the park or village due to religious preference (predominantly Muslim communities). However, bush pigs (*Potaochoerus larvatus*), native to the Gombe forest habitat, are common in Kasekela but have not been observed frequently in Mitumba forest. Thus bush pigs may serve as the reservoir host for *C*. *suis* in this system. Chimpanzees may be infected through contact with this animal (i.e., chimpanzees occasionally consume bush pigs) or by indirect contact with infective feces on the forest floor or the contamination of shared water sources. This putative pathway of transmission is supported by the fact that *C*. *suis* was not recovered in the Mitumba chimpanzee community, the baboons, domesticated livestock or village inhabitants. *C*. *suis* has zoonotic potential having been previously identified in an HIV+ patient in Lima, Peru, [54] and from patients in Henan, China and England [55,56]. Similarly, Salyer et al [28] found that while *Cryptosporidium* may be transmitted frequently among domesticated animals, humans and wildlife in areas of overlap, there may be host-parasite specific dynamics that occur in the absence of these regular interactions creating separate transmission cycles.

The appearance of the *C*. *hominis* species and its subtype IfA12G2C among the humans, baboons and chimpanzee communities demonstrates the zoonotic transmission potential of this parasite species among these closely related host species and points to a dominance of anthropozonotic transmission in this system. This subtype has been previously reported among captive olive baboons in Kenya [57], and is prevalent in humans in Africa [58]. Additionally, common primate behaviors may increase the likelihood for animal to human transmission. For example, baboons raid camp food reserves and homes, potentially transmitting etiologic agents via infected feces to humans. The Mitumba chimpanzees are reported to raid agricultural fields just outside the park boundaries, which can transmit diseases from the potentially contaminated feces of livestock or exposed human sewage.

Our results are based on small sample sizes, that if increased could alter the frequency and predictions of *Cryptosporidium* subtypes, infection and illness. The finding of *C*. *suis* in the Kasekela chimpanzee community is novel. We presume these chimpanzees experienced cross-species transmission from bush pigs in Gombe forest, because domesticated pigs are absent from the area. Unfortunately, we do not have access to fecal specimens from the local bush pig population to compare to specimens recovered from Kasekela chimpanzees. Despite the high overlap observed between people and livestock in villages in this region, our results suggest that the transmission dynamics of *Cryptosporidium* for humans and livestock are distinct but the dominance of *C*. *hominis* in humans and non-human primates suggest the potential for cross-species transmission. Our findings highlight the complex nature of zoonotic parasite transmission and stress the need for further studies in similar systems.

## Supporting Information

S1 Checklist STROBE Checklist. (PDF)
