# Correction: Epidemiology and Molecular Characterization of *Cryptosporidium* spp. in Humans, Wild Primates, and Domesticated Animals in the Greater Gombe Ecosystem, Tanzania

**DOI:** 10.1371/journal.pntd.0003650

**Published:** 2015-03-31

**Authors:** The PLOS Neglected Tropical Diseases Staff

The fifth and sixth authors’ names are spelled incorrectly. These two authors’ names are combined into one name. The correct spelling of the fifth author’s name is Dawn M. Roellig, and the correct spelling for the sixth author’s name is Anthony Collins. The author affiliation for Dawn M. Roellig is #2, Division of Foodborne, Waterborne, and Environmental Diseases, Centers for Disease Control and Prevention, Atlanta, Georgia, United States of America. The author affiliation for Anthony Collins is #5, The Jane Goodall Institute, Kigoma, Tanzania. The correct citation is: Parsons MB, Travis D, Lonsdorf EV, Lipende I, Roellig DM, Collins A, et al. (2015) Epidemiology and Molecular Characterization of *Cryptosporidium* spp. in Humans, Wild Primates, and Domesticated Animals in the Greater Gombe Ecosystem, Tanzania. PLoS Negl Trop Dis 9(2): e0003529. doi:10.1371/journal.pntd.0003529
